# Antimicrobial activity of a silver-microfibrillated cellulose biocomposite against susceptible and resistant bacteria

**DOI:** 10.1038/s41598-020-64127-9

**Published:** 2020-04-29

**Authors:** Javier Alberto Garza-Cervantes, Gricelda Mendiola-Garza, Eduardo Macedo de Melo, Tom I. J. Dugmore, Avtar S. Matharu, Jose Ruben Morones-Ramirez

**Affiliations:** 1 Universidad Autónoma de Nuevo León, UANL. Facultad de Ciencias Químicas. Av. Universidad s/n. CD. Universitaria, 66455 San Nicolás de los Garza, NL México; 2 Centro de Investigación en Biotecnología y Nanotecnología, Facultad de Ciencias Químicas, Universidad Autónoma de Nuevo León. Parque de Investigación e Innovación Tecnológica, Km. 10 autopista al Aeropuerto Internacional Mariano Escobedo, 66629 Apodaca, Nuevo León México; 3 Institute of Bio- and Geosciences 1 (IBG-1): Biotechnology, Forschungszentrum Jülich GmbH, 52425 Jülich, Germany; 4 Green Chemistry Centre of Excellence, Department of Chemistry, University of York, YO10 5DD York, England United Kingdom

**Keywords:** Biotechnology, Microbiology, Chemistry, Nanoscience and technology

## Abstract

Antibiotic Microbial Resistance (AMR) is a major global challenge as it constitutes a severe threat to global public health if not addressed. To fight against AMR bacteria, new antimicrobial agents are continually needed, and their efficacy must be tested. Historically, many transition metals have been employed, but their cytotoxicity is an issue and hence must be reduced, typically by combination with organic polymers. Cellulose of natural origin, especially those derived from unavoidable residues in the food supply chain, appears to be a good capping agent for the green synthesis of silver nanoparticles. Herein, we describe a green synthesis method to produce a novel biocomposite, using ascorbic acid as reducing agent and microfibrillated cellulose as a capping agent and demonstrate this material to be an efficient antimicrobial agent. Silver nanoparticles were obtained in the cellulose matrix with an average size of 140 nm and with antimicrobial activity against both sensitive and resistant Gram positive (using 1500 ppm) as well as sensitive and resistant Gram negative (using 125 ppm) bacteria. Also, an inverted disk-diffusion methodology was applied to overcome the low-solubility of cellulose compounds. This novel silver nanoparticle-cellulose biocomposite synthesized by a green methodology shows the potential to be applied in the future development of biomedical instruments and therapeutics.

## Introduction

Since Fleming discovered penicillin, pharmaceutical companies began producing antibiotics on a large-scale in the so-called “antibiotic gold-era”, making it easier to fight bacterial infections^1,2^. Unfortunately, the misuse of these antimicrobial compounds created a selective pressure in favor of antibiotic resistant bacteria leading to an overall increase of antimicrobial resistance (AMR)^3,4^. The World Health Organization (WHO) have campaigned against the threat of AMR with initiatives such as the Global Antimicrobial Resistance Surveillance System (GLASS); Global Antibiotic Research and Development Partnership (GARDP), and the Interagency Coordination Group on Antimicrobial Resistance (IACG). Thus, the search for new/alternative antimicrobial agents is a global grand challenge^5^.

Transition metals and their compounds, for example, silver and silver salts, are among the most studied alternatives to fight sensitive and resistant bacteria^6,7^. Metal nanoparticles, especially silver nanoparticles, have been used for a variety of biomedical applications^8^ like antimicrobial, antibiofilm activity, larvicidal and insecticidal effects, and anticancer activity^9–12^. Nevertheless, the use of such metals in therapeutic agents is limited by their toxicity on eukaryotic cells. To overcome their cytotoxic behavior, combinatorial formulations of transition metals have been proposed^13–15^, such as the use of microbial extracts or biopolymers as capping agents of metal nanoparticles (NPs) to obtain biocomposites^16–19^. Biocomposites, as well as metal nanoparticles, are commonly synthesized using green methodologies^20–26^. These methodologies are eco-friendly, eliminate the use of toxic chemicals, and allow the use of biocomposites in a variety of biological applications^27^. The typical capping agents employed on the synthesis of biocomposites are long-chain hydrocarbons, polymers, or co-polymers.

Herein, the inherent hydroxyl functionality of cellulose is considered in the preparation of a novel silver nanoparticle biocomposite. There is no shortage of cellulose as it is the most abundant biopolymer on the planet, and is also readily available from waste residues such as the unavoidable food supply chain waste. One of the more recent advances in food waste processing is the use of microwave technology as an alternative to conventional heating means. The use of microwaves to valorize citrus residues has received much attention in the past decade and has successfully been shown to produce materials such as limonene, pectin, and cellulose^28^.

Cellulose is a long-chain homopolymer comprising repeating β-(1-4)-glycosidic bonds. Its abundance, renewability, low toxicity, biocompatibility, and ease of biodegradation makes it a promising and exciting biomaterial for use in biomedical applications, namely, biocomposites to combat AMR. Different types of cellulose with distinct morphological and mechanical differences are available for composites formulation, such as vegetable cellulose (VC), bacterial cellulose (BC), and nanofibrillated cellulose (NFC)^29^. From these, BC has increased in popularity due to its high purity, crystallinity, and mechanical stabilization. However, BC production is very complicated and expensive due to high culture media costs and low rates of production^30,31^. Therefore, greener processes for industrial-scale production of cellulose fibers are under receiving increasing attention. Promising results are being obtained in the biomedical field for optimum wound dressings by enhancing *in vivo* skin repair in less than 14 days as reported by Singla *et al*. 2017 where cellulose nanocrystals from bamboo, as an alternative for BC, were obtained and functionalized with green AgNPs^32^. Thus, the use of cellulose in the synthesis of NP biocomposites could increase scalability, decrease production costs, reduce reaction time and increase safety^33,34^

Meanwhile, Life Cycle studies estimate that the increased energy efficiency from microwave technology has the potential to reduce many environmental impacts (e.g., climate change, ozone depletion, eutrophication, etc.) by ~75%^35^. The use of microwaves to produce cellulose from citrus peel can tune the properties of the resulting fibers according to processing temperature^36^. However, whilst the different chemical and physical properties of the cellulose have been established, there have been no studies on how these function in antimicrobial applications as of yet. Herein, AgNPs biocomposite synthesized using ascorbic acid as a reducing agent and microfibrillated cellulose, derived from orange peel waste, as a capping agent for the first time is here reported. Also, its antimicrobial activity against sensitive and resistant bacteria is also explored using an inverse disk diffusion method proposed here to the use of low water solubility compounds, like cellulose compounds.

## Materials and Methods

### Materials

Microfibrillated cellulose (hereafter called cellulose) from orange peel waste was obtained via acid-free microwave hydrothermal treatment at 120 °C, as reported by de Melo *et al*.^36^. Ascorbic acid and AgNO_3_ were purchased from Jalmek, Mexico. HCl (12 M) and NaOH were purchased from Desarrollo de especialidades químicas S.A de C.V., Mexico. Müller-Hinton broth was purchased from DIFCO, USA. Agar was purchased from BD Bioxon, Mexico.

### Bacterial strains

The bacteria strains used in this work were *Escherichia coli* ATCC 11229, *Staphylococcus aureus* ATCC 6538, *Pseudomonas aeruginosa* ATCC 27853, and two clinical isolates proportioned kindly by Hospital San José (Monterrey, Nuevo León, México) multidrug resistant *Staphylococcus aureus* and *Pseudomonas aeruginosa*.

### Synthesis of silver-cellulose composite

A mixture of orange peel cellulose (5000 ppm) and ascorbic acid (4%w/v) contained in a 50 mL tube was adjusted to pH 10 with NaOH and HCl and heated to 60 °C under constant agitation (600 rpm). To this, the necessary volume of AgNO_3_ (100 mM) was added until the 40 mL mark was achieved thus giving a mixture comprising cellulose (1000 ppm), ascorbic acid (1%) and AgNO_3_ (10 mM). The resultant nanoparticle composite was isolated by centrifugation (12 000 rpm for 15 min), washed (3 x ultrapure water at 12 000 rpm for 15 min), dried (SPD2010 SpeedVac, ThermoFisher Scientific, USA) for a total of 5 h, heating 1 h at 45 °C, and stored for further experiments.

### Characterization of silver-cellulose composite

The synthesis of silver nanoparticles in the cellulose was observed by UV-Vis spectrophotometry (from 300–600 nm), scanning electron microscopy (SEM), and transmission electron microscopy (TEM)^36^. For SEM, samples were dried and covered with gold/palladium (around 4 nm thickness) then analyzed on a JEOL JSM-7600F SEM. TEM images of cellulose were acquired using a TEM Tecnai 12 BioTWIN (manufactured by FEI) coupled to a SIS Megaview 3 camera at acceleration voltage of 120 kV. Before the analysis, diluted samples (0.2% aqueous) were sonicated for 30 min using an ice-cold ultrasound bath (output of 1200 W). Drops of the sample (about 8 μL) were left on the grid for 5 min, then negatively stained with 1% uranyl acetate and finally glow discharged. Copper grids with a formvar/carbon support film were used.

### Antimicrobial effect of silver-cellulose composite

Antimicrobial activity was assessed culturing the appropriate bacteria (*E. coli, P. aeruginosa*, resistant *P. aeruginosa, S. aureus, and* resistant *S. aureus*) in agar mixed with the synthesized composite. A certain amount of composite was taken to achieve concentrations of 2000, 1500, 1000, 500, 250, 125, 62, and 31 ppm in 50 mL of Müller-Hinton agar (Müller-Hinton broth with 2% of agar), sonicated until complete dispersion and emptied into sterile Petri dishes. From an overnight culture (16 h, 150 rpm at 37 °C) of each strain, 200 µL were transferred to a tube with fresh media and incubated at 37 °C (150 rpm) until an optical density at 600 nm (OD_600_) of 0.2 ± 0.02 was reached. Then, a 1:200 dilution was made to achieve a cellular concentration of ~1×10^5^ CFU/mL, and 10 µL of culture was plated in every concentration Petri dish and incubated at 37 °C for 20 h. After the incubation, the growth of bacteria was observed in every concentration plate. All experiments were conducted in triplicate.

## Results and Discussion

### Synthesis and characterization of silver-cellulose composite

**Figure 1:**
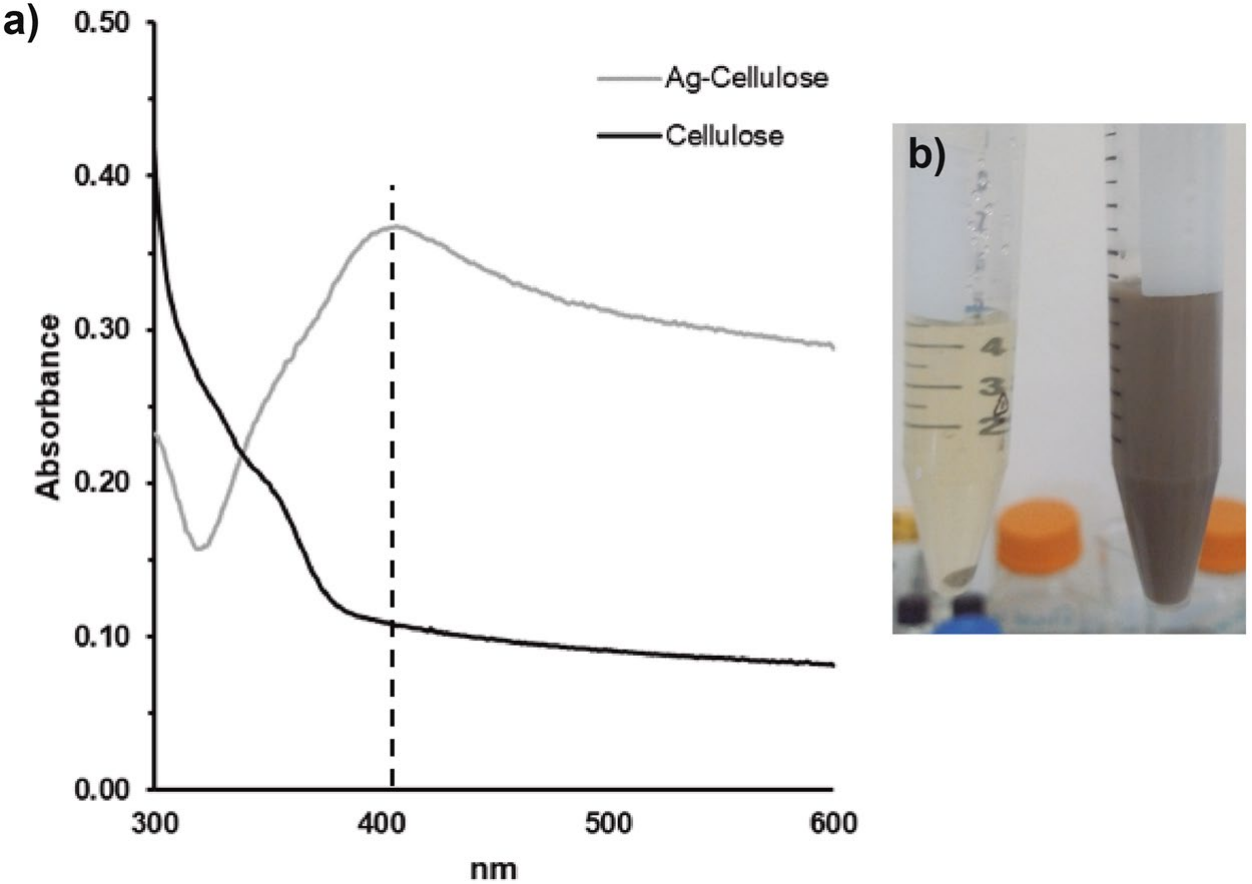
UV-Vis spectrum of silver nanoparticles synthesized in the cellulose matrix. (**a**) silver nanoparticle peak of 407 nm; (**b**) color change of the AgNPs synthesis.

The synthesis of metal nanoparticles was carried out through a green methodology were either none or reduced environmentally toxic agents were employed. The synthesis used an eco-friendly reducing agent, ascorbic acid, commonly used in the synthesis of metal nanoparticles^25,37,38^. The successful formation of silver nanoparticles (AgNPs) was observed using UV-Vis spectrophotometry (Fig. 1a). This was evidenced by the formation of the characteristic surface plasmon resonance (SPR) absorbance band centered at 407 nm, commonly reported around 420 nm^39–41^, the lack of a band in the cellulose spectrum and visually, via color change (light yellow to black to grey), indicative of the redox reaction between the Ag salt and ascorbic acid (Fig. 1b). This color was persistent in the AgNPs-cellulose compound during three months of realization of the remaining experiments, suggesting that the cellulose used in this work provides good stability to the synthesized AgNPs^42^.

**Figure 2:**
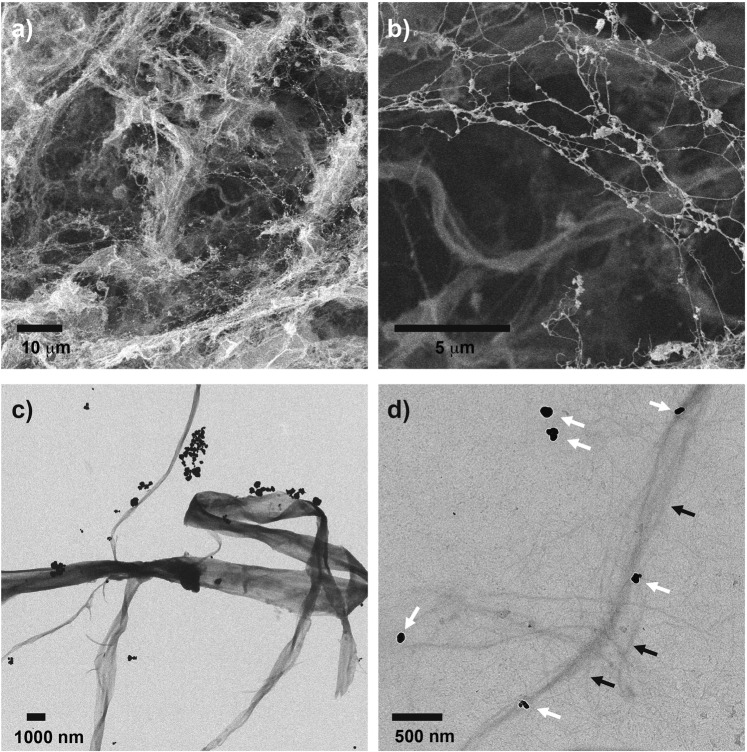
Electron microscopy images of AgNPs in cellulose. (**a**) and (**b**) SEM, (**c**,**d**) TEM images of the AgNPs synthesized in the cellulose matrix, white arrows point AgNPs, and black arrows point defibrillated cellulose.

The formation of AgNPs within a defibrillated cellulosic matrix was further evidenced by SEM (Fig. 2a,b) and TEM (Fig. 2c,d). These images show that the AgNPs obtained are spherical and caped with the cellulose matrix. Several works reported the obtention of spherical AgNPs when ascorbic acid was used as a reducing agent, where the size of the AgNPs varies depending on the capping agent, pH value, and reaction time used in the synthesis^43–45^. Here, the capping effect induced by the cellulosic substrate gave an average size of 140.79 ± 85.41 nm AgNPs. These physical characteristics of the material are due to the specific synthesis conditions established and reported in this work reaction regarding time and pH values^46^. As reported^36^, the cellulose used in this work has a high amount of hydroxyl groups as well as substantial intra- and intermolecular hydrogen bonding interactions, characteristic of cellulose compounds^47^. These chemical groups could be involved in the stabilization of the AgNPs by anchoring silver ions into the cellulose fibers and stabilizing the AgNPs due to the interaction between cellulose hydrogen bonds and the metal nanoparticle^25,48^. Also, the use of microwave technology to produce the cellulose also presents the opportunity to reduce the environmental impact of its production significantly compared to other materials in the field^36^.

### Antimicrobial effect of the Ag-cellulose composite

The antimicrobial activity against a selected group of the clinically relevant ESKAPE pathogens^49–52^ (*Staphylococcus* and *Pseudomonas* resistant strains) is here reported for a synthesized metal biocomposite. To assess the antimicrobial activity of certain compounds, the Minimum Inhibitory Concentrations (MIC) obtained from the disk diffusion method is a well-accepted parameter, but this method depends directly on the facile diffusion of the antimicrobial agent in the agar. Cellulose compounds, like the AgNP composite synthesized here, are known to have low solubility in water, and therefore in agar as well, in contrast to AgNPs capped with water-soluble compounds, which exhibit favorable antimicrobial activity by this methodology^53,54^. Besides, the diffusion of silver, in silver-containing materials, could be limited by the composition of the media used for the determination of inhibition zones^55^. Thus, an inverse disk diffusion method was used, which involves dispersing the biocomposite in the agar and placing the bacteria as “disks” at a known concentration.

**Figure 3:**
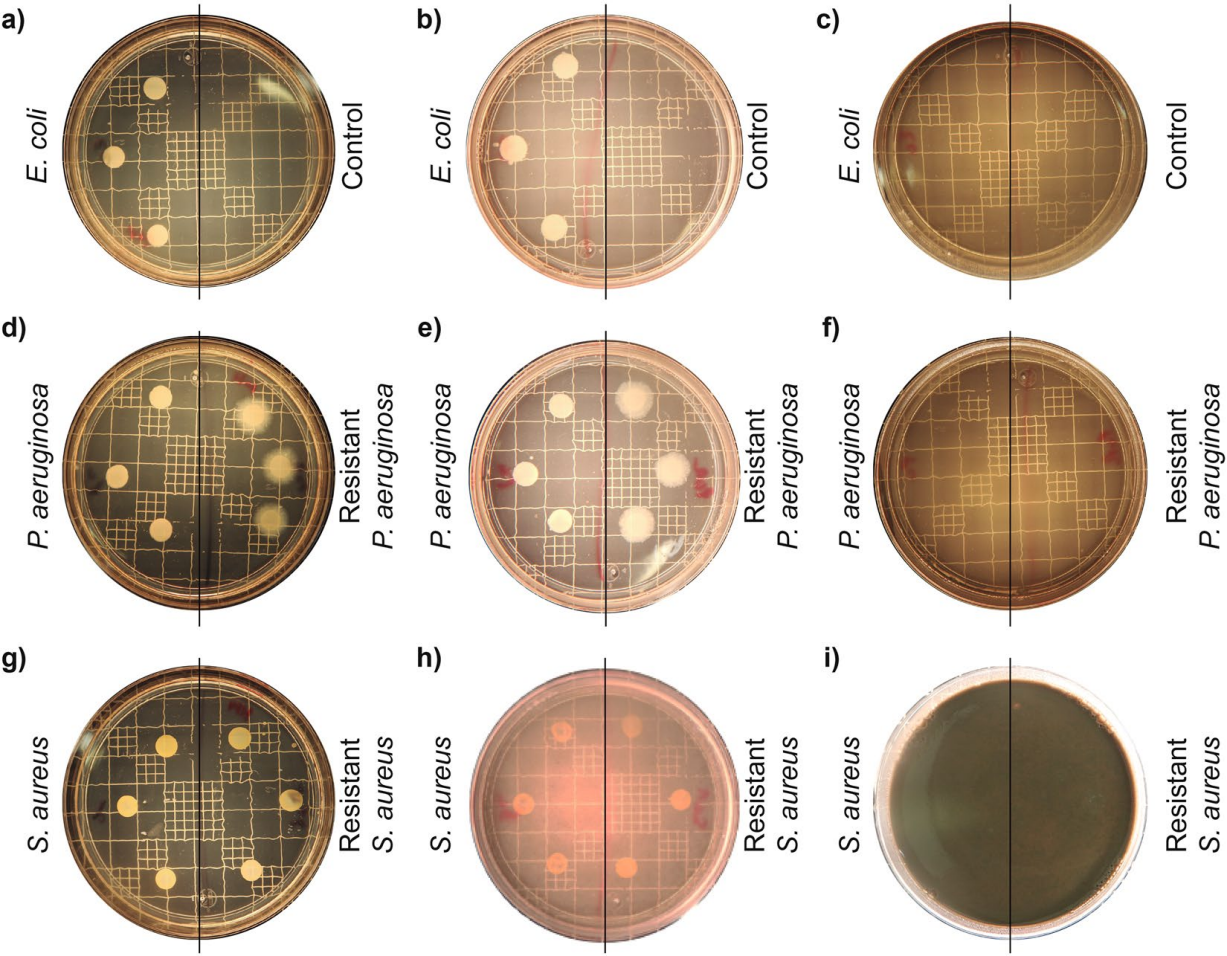
Growth inhibition caused by AgNPs composite. (**a**) *E. coli* untreated control plate, (**b**) *E. coli* at 62 ppm, (**c**) *E. coli* at 125 ppm, **(d**) ATCC and resistant *P. aeruginosa* control plate, (**e**) ATCC and resistant *P. aeruginosa* at 62 ppm, (**f)** ATCC and resistant *P. aeruginosa* at 125 ppm, (**g**) ATCC and resistant *S. aureus* control plate, (**h**) ATCC and resistant *S. aureus* at 1000 ppm and (i) ATCC and resistant *S. aureus* at 1500 ppm of AgNP composite.

The MICs obtained are reported in Table 1. Both Gram positive strains (*S. aureus* and *resistant S. aureus*) were inhibited at 1500 ppm, whereas the Gram negative strains (*E. coli, P. aeruginosa* and *resistant P. aeruginosa*) needed only 125 ppm for complete inhibition. These behaviors have been reported for bare and coated AgNPs and could be due to the differences in the cell membrane as Gram positive bacteria have a thicker peptidoglycan layer that could prevent the penetration of our biocomposite into the cell cytoplasm^56–58^. Moreover, recent reports of AgNPs-cellulose biocomposites against *E. coli* have shown good inhibition zones values of 15–22.5 mm reliant on the aq. AgNO_3_ solution concentration which varied from 4 mM to 250 mM (from 430 to 26 000 ppm of Ag)^59–61^. Similarly, reports of AgNPs-cellulose compounds against *S. aureus* have shown good inhibition zones, using aq. Ag concentrations from 1 mM to 79 mM (from 107 to 8521 ppm)^62–64^. Taking a total reduction of the AgNO_3_ used, the Ag:cellulose ratio in 1 mg of the synthesized compound is calculated to be 0.519:0.418 mg. Thus, the MICs of 125 ppm, obtained for the Gram negative strains, and 1500 ppm, obtained for the Gram positive strains, would have a total of 64 and 778 ppm of silver, respectively. Thereby, our Ag-cellulose composite shows an efficiency five times higher set off against the most optimum clear zone value obtained for Gram negative strains and up to 10 times higher for Gran positive strain according to the literature. This composite represents an attractive alternative to the treatment of infectious diseases caused by bacteria, especially the bacteria strains used in this work, as they are included in the ESKAPE groups, which, as mentioned above, represent an important clinical threat^49^. Figure 3 shows the bacterial growth of the untreated control, a sub-inhibitory concentration, and the MIC. As AgNPs concentration increases, agar medium color intensifies to a darker grey. Thence the lowest tonality is present at 62 ppm and the darkest plate at 1500 ppm.

**Table 1:** Minimum inhibitory concentration of the synthesized AgNP composite.

Microorganism	MIC (ppm)
*Escherichia coli* ATCC 11229	125
*Pseudomonas aeruginosa* ATCC27853	125
Resistant *Pseudomonas aeruginosa*	125
*Staphylococcus aureus* ATCC 6538	1500
Resistant *Staphylococcus aureus*	1500

## Conclusions

A novel biocomposite containing AgNPs was synthesized using defibrillated cellulose. The methodology proposed in this work to determine the antimicrobial activity was relevant as the biocomposite was homogeneously dispersed in the agar plate, overcoming the low diffusion it would have if placed in conventional disks. Also, the novel biocomposite showed antimicrobial activity against reference bacteria strains as well as against relevant clinical multidrug-resistant strains and showed a stronger antimicrobial activity against Gram negative than Gram positive bacteria. Based on the results reported in this manuscript, this biocomposite can be considered for further studies regarding its application in biomedical fields.
